# Biosolid-Amended Soil Enhances Defense Responses in Tomato Based on Metagenomic Profile and Expression of Pathogenesis-Related Genes

**DOI:** 10.3390/plants10122789

**Published:** 2021-12-16

**Authors:** Evangelia Stavridou, Ioannis Giannakis, Ioanna Karamichali, Nathalie N. Kamou, George Lagiotis, Panagiotis Madesis, Christina Emmanouil, Athanasios Kungolos, Irini Nianiou-Obeidat, Anastasia L. Lagopodi

**Affiliations:** 1 Institute of Applied Biosciences, Centre for Research and Technology Hellas, 57001 Thessaloniki, Greece; estavrid@certh.gr (E.S.); ikaramichali@certh.gr (I.K.); glagiotis@certh.gr (G.L.); pmadesis@uth.gr (P.M.); 2 Laboratory of Genetics and Plant Breeding, School of Agriculture, Forestry and Natural Environment, Aristotle University of Thessaloniki, 54124 Thessaloniki, Greece; 3 School of Civil Engineering, Aristotle University of Thessaloniki, 54124 Thessaloniki, Greece; iogianna@civil.auth.gr (I.G.); kungolos@civil.auth.gr (A.K.); 4 Laboratory of Plant Pathology, School of Agriculture, Forestry and Natural Environment, Aristotle University of Thessaloniki, 54124 Thessaloniki, Greece; ngkamou@agro.auth.gr; 5 Laboratory of Molecular Biology of Plants, School of Agricultural Sciences, University of Thessaly, 38221 Volos, Greece; 6 School of Spatial Planning and Development, Aristotle University of Thessaloniki, 54124 Thessaloniki, Greece; chemmanouil@plandevel.auth.gr

**Keywords:** biosolid leachates, sludge, *S. lycopersicum* L., *Fusarium oxysporum*, PR-related genes, defense-related proteins, soil bacteria communities, 16S sequencing, biocontrol

## Abstract

Biosolid application is an effective strategy, alternative to synthetic chemicals, for enhancing plant growth and performance and improving soil properties. In previous research, biosolid application has shown promising results with respect to tomato resistance against *Fusarium oxysporum* f. sp. *radicis*-*lycopersici* (Forl). Herein, we aimed at elucidating the effect of biosolid application on the plant–microbiome response mechanisms for tomato resistance against Forl at a molecular level. More specifically, plant–microbiome interactions in the presence of biosolid application and the biocontrol mechanism against Forl in tomato were investigated. We examined whether biosolids application in vitro could act as an inhibitor of growth and sporulation of Forl. The effect of biosolid application on the biocontrol of Forl was investigated based on the enhanced plant resistance, measured as expression of pathogen-response genes, and pathogen suppression in the context of soil microbiome diversity, abundance, and predicted functions. The expression of the pathogen-response genes was variably induced in tomato plants in different time points between 12 and 72 h post inoculation in the biosolid-enriched treatments, in the presence or absence of pathogens, indicating activation of defense responses in the plant. This further suggests that biosolid application resulted in a successful priming of tomato plants inducing resistance mechanisms against Forl. Our results have also demonstrated that biosolid application alters microbial diversity and the predicted soil functioning, along with the relative abundance of specific phyla and classes, as a proxy for disease suppression. Overall, the use of biosolid as a sustainable soil amendment had positive effects not only on plant health and protection, but also on growth of non-pathogenic antagonistic microorganisms against Forl in the tomato rhizosphere and thus, on plant–soil microbiome interactions, toward biocontrol of Forl.

## 1. Introduction

The fungus *Fusarium oxysporum* f.sp. *radicis*-*lycopersici* (Forl) is a destructive pathogen limiting crop productivity and causing significant losses in commercial tomato production worldwide [1,2]. Forl is a saprotrophic soilborne pathogen causing tomato foot and root rot disease (TFFR) [2] by intense colonization of the root hair zone and especially the crown of the plant [1]. Due to the resistant nature of all formae speciales of *F. oxysporum*, this fungus is extremely difficult to control with synthetic fungicides [2]. Furthermore, synthetic fungicides may also affect beneficial soil microbiota and may accumulate in the food chain. Therefore, developing alternative, more efficient methods to control Forl is necessary [3,4]. To date, several methods have been used to limit the spread of Forl in field, and greenhouse conditions, such as the use of resistant tomato hybrids and rootstocks, as well as soil disinfection [5], which is a rather expensive practice as it must be repeatedly applied, leading in turn to the increase in hazardous inputs in agriculture.

Alternative methods have been proven to trigger mechanisms of disease control in plants, such as the addition of beneficial antagonistic microorganisms applied in tomato rhizosphere [6], or of a suitable soil conditioner containing beneficial microorganisms, such as green waste compost mixtures or sewage sludge [7,8,9]. Additionally, soil properties and improvement of plant vitality and growth conditions have also been considered as an important factor for altering plant rhizosphere microorganism composition and thus strengthening plant defense [10]. Studies have shown that fertilization with calcium-rich soil amendments, may enhance soil fertility, strengthen the plant defense systems and thus, suppress pathogen infections [11]. This is facilitated mainly through changes in the soil properties, such as the increase in the soil pH, which favors soil microorganisms (actinomycetes and bacteria) that thrive at such pH values and are competitive with Forl.

Recently, Giannakis et al. [12] reported that sludge-based biosolids enhance tomato growth and reduce TFFR severity. Biosolids may benefit the plant’s health by improving the soil properties and enhancing the diversity of the rhizosphere bacterial community [13,14]. Soil microbiota play a key role in soil suppressiveness [15], and studies have shown that they are involved in disease suppression [16,17]. Therefore, microbial community abundance, richness, evenness, and diversity have been identified as key factors involved in community functioning, soil health, and plant productivity [8]. Studies have shown that changes in the rhizosphere microbial community may affect the plant’s resistance to different formae speciales of *F. oxysporum* [18,19,20]. Additionally, there is growing evidence that greater disease suppression is induced by a consortium of plant beneficial bacteria rather than individual strains [21]. However, identifying specific bacterial species and the underlying response mechanisms triggered by the application of biosolids against TFFR have yet to be determined. Hence, further insight into the interactions between the pathogen and the potentially suppressive soil microbiota will provide further insight into the mechanisms underlying the plant–microbe interactions against Forl and how biosolid application may impact this relationship.

Considering that *F. oxysporum* challenges not only tomato, but also several Solanaceae species of great agronomic and economic importance, the interaction between tomato and *Fusarium* has been extensively studied as a model pathosystem for disease resistance response [22]. It is therefore important to further investigate the effects of soil amendment methods on plant–pathogen systems and especially their effect on plant resistance mechanisms through monitoring gene regulation and microbiome diversity in the rhizosphere. The defense system of plants against pathogens involves regulation of gene expression, activation of signaling pathways, hormone balancing, and synthesis of defensive metabolites [23]. An investigation of the transcriptomic profile of tomato plants infected with *F. oxysporum* f. sp. *lycopersici* has revealed up-regulated gene expression related to plant response mechanisms and plant–pathogen interactions, mainly associated with maintenance of cellular structures and homeostasis [24]. Recently, Kamou et al. [4] reported induction of defense gene expression in tomato challenged with Forl. More specifically, salicylic acid (SA)-related genes such as *PR-1a* and *GLUA*, together with jasmonic acid (JA)-related ones such as the *CHI3*, were overexpressed in the presence of the pathogen but also after inoculation with beneficial *P. chlororaphis* ToZa7, revealing induction of variable defense mechanisms. Interestingly, studies have demonstrated the important role of the defense-related phytohormones SA and JA as modulators of the rhizosphere microbiome assembly of plants, such as *Phaseolus vulgaris* and *Arabidopsis thaliana* [25,26]. Moreover, plant’s ability to recruit a community of beneficial microbiota and exploit protective rhizosphere processes to their advantage is genotype dependent [27,28].

As a follow-up to the report of Giannakis et al. [12] on the beneficial effects of sludge-based biosolids on tomato growth and TFFR severity, in the present work, we have investigated the plant–pathogen interactions at a molecular level and how they are affected by the addition of biosolids in the soil. More specifically, it was examined whether biosolids: (i) could act in vitro as an inhibitor of growth and sporulation of Forl, (ii) could induce gene expression related to plant response against pathogens in tomato, and (iii) would provide a beneficial substrate for the growth of non-pathogenic antagonistic microorganisms against Forl in the tomato rhizosphere. To achieve this, the relative expression analysis of genes related to defense mechanisms in tomato was analyzed; 16S sequencing analysis of the soil substrates was also performed to determine the genetic diversity and functions of microbial (bacteria and archaea) communities present in the soil substrates, which may have a beneficial effect to the plant and/or suppress the pathogen. This work may elucidate the mechanisms through which biosolid addition enhances plant resistance against pathogens. Furthermore, it is expected to contribute to deciphering the effect of biosolids on soil microbial community to sustainably suppress TFFR disease in tomato crops.

## 2. Results

### 2.1. Growth and Sporulation of Fusarium oxysporum f. sp. radicis-lycopersici

**Figure 1:**
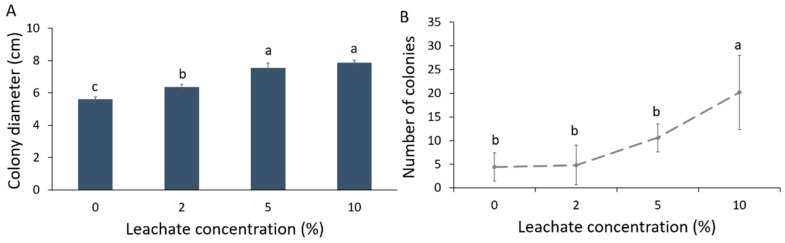
Growth and sporulation of *Fusarium oxysporum* f. sp. *radicis-lycopersici* (Forl) at 0, 2, 5, and 10% biosolid leachate concentrations in PDA. (**A**) Mean colony diameter; (**B**) Mean sporulation. Different letters indicate statistically significant differences between the treatments according to Tukey’s post hoc test (*p* < 0.05).

Colony diameter (cm) of Forl increased in the different mixtures of biosolid leachates with PDA compared to the control (Figure 1A). This increase was greater with elevating leachate concentration. As such, the concentrations of 5 and 10% leachate showed the largest colony diameter (an increase of 40% was noted in relation to control) followed by the 2% leachate, where an increase of 28% in colony diameter was noted in relation to control. Sporulation was also significantly affected by the biosolid leachate; the number of fungal spores increased significantly at 10% concentration of leachate (Figure 1B). Hence, the number of conidia produced per cm^2^ of colony was increased with the increase in the leachate concentration (Table 1).

### 2.2. Gene Expression Analysis

**Figure 2:**
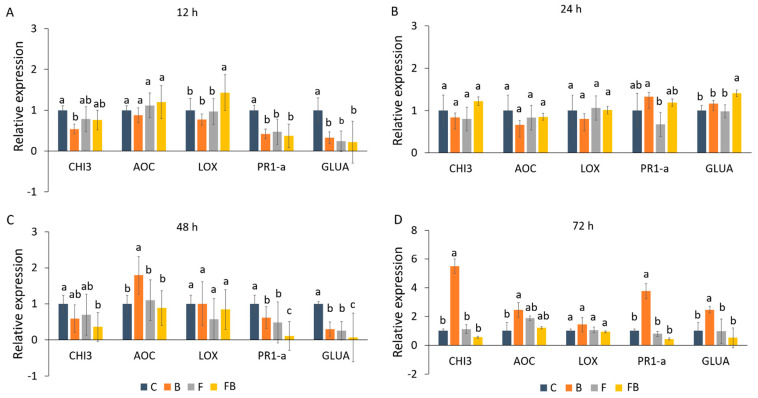
(**A**–**D**) Relative gene expression analyses of defense-related genes *GLUA*, *CHI3*, *PR1-a*, *LOX*, and *AOC*, in tomato plant leaves in (**A**) 12 h, (**B**) 24 h, (**C**) 48 h, and (**D**) 72 h post inoculation in control (C), biosolid−enriched treatment (B), Forl inoculation (F), and Forl inoculation with biosolid application (FB). Different letters indicate statistically significant differences between the treatments according to Tukey’s post hoc test (*p* < 0.05).

**Figure 3:**
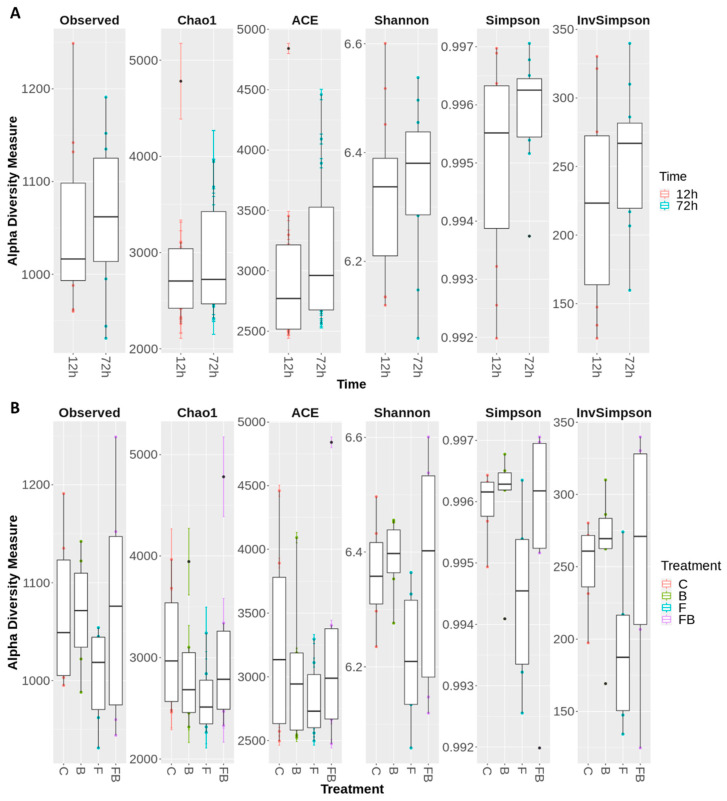
Boxplot demonstrating the range and the distribution of the of OTUs, evaluated using the α-diversity indices observed richness, Chao1, ACE, Shannon, Simpson, and InvSimpson, (**A**) 12 and 72 h after inoculation with *Fusarium oxysporum* f. sp. *radicis-lycopersici*; (**B**) for the treatments for the treatments control (C), biosolid−enriched treatment (B), Forl inoculation (F), and Forl inoculation with biosolid application (FB). Values represent the pooled mean of three replicates.

Expression patterns of defense-related genes (*GLUA*, *CHI3*, *PR1-a*, *LOX*, and *AOC)* following application of biosolid and inoculation with Forl were analyzed using RT-qPCR (Figure 2). The induction patterns were evaluated in tomato plants at 12, 24, 48, and 72 h after inoculation. As internal control the reference gene *β-actin* was used. A significant upregulation of *LOX* was observed at 12 h in response to Forl inoculation and the addition of biosolid (FB) as compared to the treatments C, F, and B (Figure 2A). Nevertheless, in the later time points, *LOX* was not significantly induced (Figure 2B–D). Interestingly, at 12 h an increase in the *AOC* expression levels was also observed after application of biosolid and Forl inoculation (FB), yet this was not significant in comparison to the other treatments (Figure 2A). However, at 12 h, no induction in *PR1-a*, *GLUA*, and *CHI3* was observed for the different treatments in relation to the control (C) (Figure 2A). At 24 h after inoculation, only *GLUA* was significantly induced in the FB treatment, whilst the *PR1-a* gene was significantly overexpressed only under biosolid application (B), yet a non-significant increase in the expression levels was also observed under FB treatment (Figure 3B). *AOC* was significantly induced at 48 and 72 h after biosolid application (Figure 2C,D). At 72 h (Figure 2D) overexpression of 2.45-, 5.5-, 3.76-, and 2.45-fold was observed for *CHI3*, *AOC*, *PR1-a*, and *GLUA*, respectively, under biosolid application (B), but not after inoculation with Forl (FB) when compared with the untreated control.

### 2.3. Characterization of Microbial Communities in the Different Soil Substrates

Based on the rank abundance curves for the top 100 OTUs (Figure S1) an even abundance was observed between the 12 and 72 h (Figure S1A). However, the treatments without the addition of biosolid, i.e., F and C, showed unevenness of abundance in soil bacterial communities due to the large difference in detected organisms, whereas in the treatments where biosolid was added (B and FB), species evenness was more homogeneous (Figure S1B).

**Figure 4:**
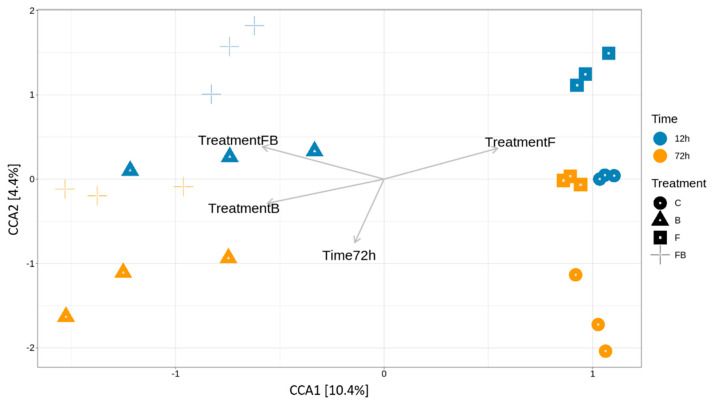
Canonical correspondence analysis (CCA) of the relative variance of OTUs in the soil samples subjected to the different control (C), biosolid−enriched treatment (B), Forl inoculation (F), and Forl inoculation with biosolid application (FB) treatments at 12 and 72 h. The biplots present the effect of biosolid application to the bacterial community abundance in the treatments with or without fungal inoculum at two different timepoints.

Microbial diversity was evaluated using the following indicators: observed richness, Chao1, ACE, Shannon, Simpson, and InvSimpson (Figure 3A and Figure 4B). The alpha diversity indices were notably increased (or did not significantly change) in soil substrate samples at 72 h, indicating that the shift pattern of the microbiome occurred at a later timepoint (Figure 3A). Respectively, treatments enriched with biosolid, regardless of the presence or absence of Forl inoculum (FB and B, respectively) showed greater microbiome diversity in *α*-diversity indices (Observed richness, Shannon, and Simpson), whilst the treatment inoculated with Forl (F) showed a lower species evenness (Figure 3B), indicating that the relative abundances of species within the community vary in distribution.

To further relate bacterial community variance to the different variables, such as the effect of time post Forl inoculation (F and FB) and biosolid application (B and FB) and the treatments, Canonical Correspondence Analysis was performed (Figure 4). The variation explained by the treatments and timepoints (constrained ordination) was 22.8% and the remaining 77.2% of the variation was explained by the unconstrained ordination (Table 2). From the eigenvalues of the constrained axes, it was observed that 45.65% of the variation is explained by the CCA1 and the 19.1% by CCA2 (Table 3). The factors responsible for most of the explained variation in the bacterial community were the treatments enriched with biosolid (B and FB), which were differentiated from those inoculated with Forl without the addition of biosolid (F) at 72 h (Table 4). Therefore, the addition of biosolid, with or without Forl inoculum, affected differently the bacterial community abundance compared to the control and Forl treatments.

**Figure 5:**
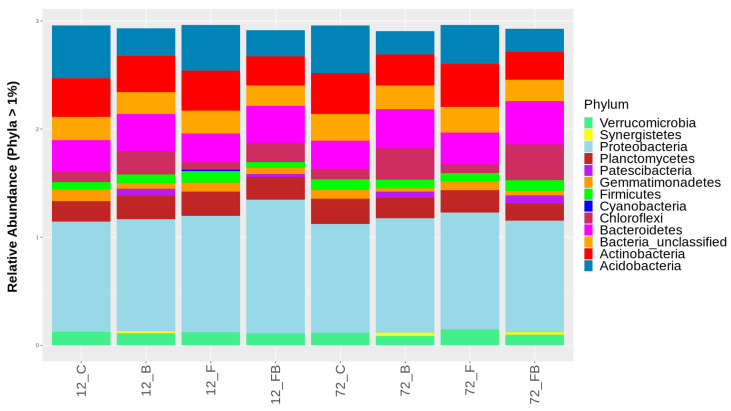
Classification of bacterial phyla based on relative abundance at rate greater than 1%, for the control (C), biosolid−enriched treatment (B), Forl inoculation (F), and Forl inoculation with biosolid application (FB) treatments, at 12 and 72 h after inoculation with *Fusarium oxysporum* f. sp. *radices-lycopersici*. The different phyla are depicted with different colors. Value is the pooled mean of three replicates.

**Figure 6:**
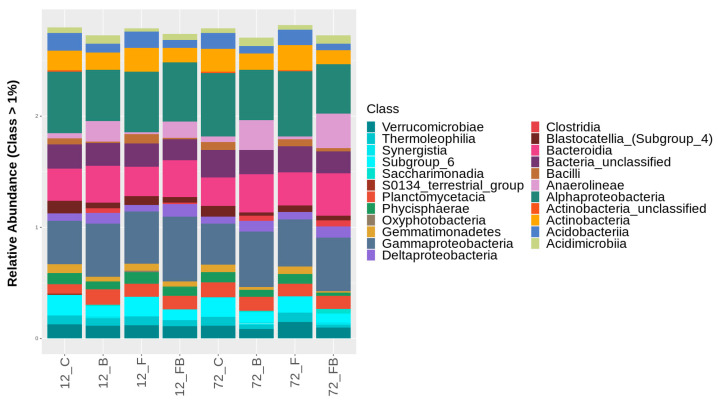
Classification of bacterial classes based on relative abundance at rate greater than 1%, for the treatments control (C), biosolid−enriched treatment (B), Forl inoculation (F), and Forl inoculation with biosolid application (FB) at 12 and 72 h after inoculation with *Fusarium oxysporum* f. sp. *radicis-lycopersici*. The different classes are depicted with different colors. Value is the pooled mean of three replicates.

Composition of bacterial populations at the phylum and class taxonomic levels were evaluated using the β-diversity analysis for each treatment and time point tested. Overall, 38 and 94 different phyla and classes, respectively, were identified across the treatments in the two timepoints, yet relative abundance of only 13 phyla and 23 classes with OTUs representation >1% are shown in Figure 5 and Figure 6, respectively. The most abundant amplicons were identified as *Proteobacteria*, *Bacteroidetes, Actinobateria, Acidobacteria, Gemmatimonadetes, Chloroflexi*, and *Planctomycetes*. The biosolid-enriched treatments (B and FB) were characterized by higher abundance of amplicons identified as *Chloroflexi* and *Bacteroidetes*, which increased with time (Figure 5, Table S1). Moreover, the phylum *Patescibacteria*, was detected only in biosolid-enriched treatments (B and FB) in both time points (Figure 5, Table S1). The phylum *Synergistetes* was initially observed in treatment B at 12 h and 72 h and in FB only at 72 h. In contrast, *Acidobacteria* and *Actinobacteria* were characterized by lower abundance in the biosolid-enriched soils (B and FB) compared to the control (C) and Forl (F) treatments (Figure 5, Table S1).

At the bacterial class level, the treatments enriched with biosolid (B and FB) showed higher relative abundances of *Gammaproteobacteria, Bacteroidia, Anaerolineae, Deltaproteobacteria*, and *Acidimicrobiia* in both time points (Figure 6, Table S2). Interestingly, *Clostridia* were detected solely in biosolid-enriched soils and increased with time. A similar trend was observed for *Saccharimonadia* and *Synergistia*, yet these were not present in FB soils at 12 h. In contrast, the classes *Alphaproteobacteria*, *Actinobacteria*, *Planctomycetacia*, *Verrucomicrobiae*, *Acidobacteriia*, *Phycisphaerae*, *Thermoleophilia*, and *Gemmatimonadetes* were found in higher abundance in control soils (C) and soils with Forl inoculum (F) compared to biosolid-enriched treatments (Figure 6, Table S2). Unclassified actinobacteria were solely detected in C and F treatments. However, in the C treatment the call abundance was reduced over time and in F treatment increased over time. The class *Blastocatellia* (Subgroup 4) showed higher abundance under C conditions and was reduced with either biosolid application and/or Forl inoculation. Soils in C and F treatments showed higher abundance of *Bacilli*, compared to the soils treated with biosolid (B) (Figure 6, Table S2). However, at 72 h, the abundance of this class increased in C treatment, yet it was reduced in B, F, and FB treatments (Figure 6, Table S2). The class *Oxyphotobacteria* was only present in F treatment and *S0134_terrestrial_group* in C treatment at 12 h (Figure 6, Table S2).

### 2.4. Predicted Functional Diversity of the Microbiome Present in the Different Soil Substrates

**Figure 7:**
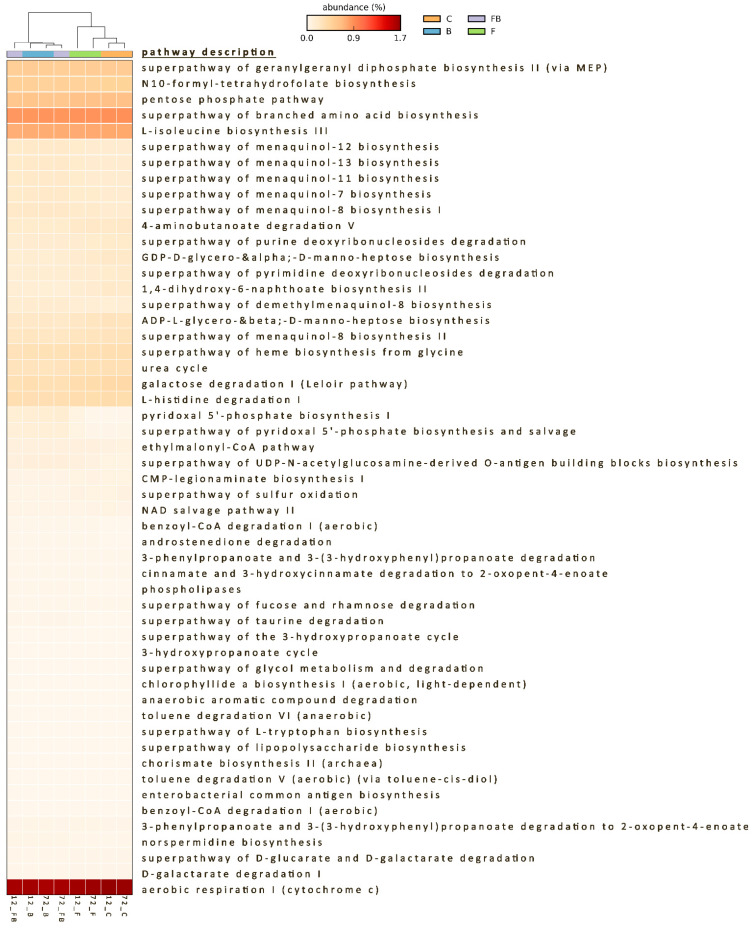
Heatmap of the predicted functional profile for the control (C), biosolid (B), Forl inoculation (F), and combination of Forl inoculation and biosolid application (FB) analyzed using STAMP software. The key shows the % relative abundances for *p*-value ≤ 0.05 and effect size >0.85 (n = 3).

The functional potential encoded by the soil microbial communities showed significant differences in the bacterial metabolic pathways among the treatments. The functional profiles from the 16S sequences revealed different metabolic capacity between the microbiome in biosolid-enriched and the non-biosolid-enriched soils (Figure S1), with the biosolid application explaining the 81.5% (PC1) of the functional variation and time only 12.2% (Figure S2). Out of 445 pathways, only 98 pathways passed the filter of *p* < 0.05 with an effect size >0.85, and 53 pathways with an effect size >0.9 (Figure 7; Table S3). Biosolid-enriched treatments B and BF showed higher abundance in sequences assigned to metabolic pathways such as carbohydrate biosynthesis (gluconeogenesis), vitamin biosynthesis (folate and vitamin B6) (Figure S3), electron carrier biosynthesis (quinol and quinone; menaquinol) (Figure S3), fermentation (pyruvate), and nucleic acid processing (Table S3). Lower abundances in sequences of B and BF treatments were attributed to pathways such as aromatic compound degradation, autotrophic CO_2_ fixation, CMP-sugar biosynthesis, fatty acid and lipid degradation, and nitrogen compound metabolism (Table S3). High abundance of sequences in biosolid-enriched soils with Forl inoculation (BF treatment) were also assigned to metabolic pathways of vitamin biosynthesis (folate transformations III) and pathways with lower abundance, such as aromatic compound biosynthesis (chorismate), fatty acid and lipid biosynthesis, and polysaccharide biosynthesis (Table S3).

Several common pathways were observed between control (C) and Forl inoculated (F) treatments, such as aerobic respiration, S-adenosyl-L-methionine biosynthesis, tetrapyrrole biosynthesis, nucleoside, and nucleotide degradation (purine), proteinogenic amino acid biosynthesis, sugar derivative degradation, sugar nucleotide biosynthesis and sugar degradation (galactose), terpenoid biosynthesis, nitrogen compound metabolism, menaquinol biosynthesis, heme b biosynthesis, and TCA cycle (Table S3). However, Forl inoculation treatment also had higher abundance of sequences attributed to carboxylate (sugar acid) and secondary metabolite (sugar derivative; sulfoquinovose) degradation (Table S3).

## 3. Discussion

Biosolids have been characterized as organic soil amendments, supplying the soil with nutrients and organic compounds and contributing to soil moisture and aeration [29,30,31]. However, there is limited research related to the molecular mechanisms associated with biosolid-elicited suppression of soilborne diseases, such as TFFR and the enhanced plant performance. In a previous work, biosolid application in the soil has been shown to enhance tomato growth and reduce the effects of Forl infection [12]. In the present work, a molecular approach was employed to provide further insight into the mechanistic effect of biosolid application in alleviating the negative impact of Forl on tomato plants. To achieve this, gene expression profiles related to plant response against pathogens coupled with 16S metagenome profile analysis were used to determine the genetic diversity and functions of bacterial communities present in the soil substrates and their potential impact on plant–pathogen relationships.

Biosolid application can enhance tomato tolerance against the Forl [12], which was mainly attributed to the indirect beneficial effects of biosolid application on biotic and abiotic factors [31]. Although growth and sporulation of Forl in the presence of biosolids were not studied in planta, it would have been expected that biosolids could suppress the growth and sporulation of Forl, thus reducing its aggressiveness against tomato. In contrast, based on the growth analysis and sporulation of Forl under in vitro conditions, herein, an increase in colony diameter, the number of fungal spores of Forl, and the number of conidia produced per cm^2^ of colony was observed in response to the increasing concentration of biosolid-PDA leachates, compared to the control. Such positive effect of biosolid leachates on the colony growth and spore production in vitro could be explained by the fact that various biοsolid leachates contained significant amounts of calcium (Ca) and magnesium (Mg) [32]. PDA used for the growth of Forl is a universally used medium providing the necessary nutrients for the growth of fungi for laboratory purposes. However, nutritional requirements in macro- and microelements vary among different fungal species [33]. Magnesium is considered a macro element necessary for enzyme activation and ATP metabolism. On the other hand, Ca is generally accepted as a micronutrient required for enzyme activity and membrane structure in fungi [33] and plays a key role in hyphal tip growth [34]. It is assumed that nutrients provided in the leachates exert positive effects on hyphal growth and production of conidia in Forl. However, this calls for further investigation, as the nutritional requirements for the improvement of growth and sporulation has not been studied in this fungus.

Nevertheless, biosolid application in soils induced an upregulation of defense-related genes in tomato plants post Forl inoculation. Increase in transcript levels of defense-related genes indicate activation of the tomato response mechanisms against the pathogen [35,36,37]. The observed increase in mean transcript levels of *LOX*, *AOC*, and *GLUA* in the biosolid-enriched soils (B and FB) as compared to the non-amended soils (C, F), indicated that biosolid application may play a key role in the early (12 and 24 h) activation of the tomato response mechanisms against Forl. Activation of the ethylene (ETH) and jasmonic acid (JA)/ETH signaling pathways even at 72 h in FB treatment may indicate an attempt to limit pathogen progression under the effect of biosolids. JA elicitor is a signaling molecule involved in various plant developmental processes and defense mechanisms [38]. The role of JA pathway in protection against Forl was confirmed in tomato after biochar application [39]. More specifically, biochar application induced upregulation of the pathways and genes associated with plant defense and growth such as JA, yet biosynthesis and signaling of the salicylic acid (SA) pathway was downregulated (Jaiswal et al., 2020). In contrast, herein, genes involved in the SA biosynthesis and signaling pathways, such as *GLUA* and *PR1-a*, were upregulated in the biosolid-enriched treatments (B and FB). However, the *PR1-a* gene was significantly upregulated only under biosolid application (B) at early time points and only at 72 h in both B and FB treatments. PR proteins are elicited in many plant species by the attack of different pathogens. Plants inoculated with Forl (F) did not show any significant upregulation of the analyzed defense-related genes, which is consistent with other studies showing a delayed induction of defense-related gene expression only 2–3 weeks post inoculation [40,41,42]. This suggests that either Forl needs to be in close association with tomato roots for upregulation of *PR1-a* to take place, that the accumulation of PR proteins requires longer period post inoculation [42], or even that the selected genotype was rather tolerant to Forl [43]. It has been shown that Forl first interacts with tomato roots 48 h after inoculation [1]. In addition, it has been shown that the developmental stage seems to play an important role in the induction of resistance genes [35].

Nevertheless, genes associated with the JA and SA biosynthesis and signaling pathways such as *CHI3, AOC, PR1-a*, and *GLUA* were upregulated 2.45-, 5.5-, 3.76-, and 2.45-fold, respectively, at 72 h in biosolid without Forl (B), but not after inoculation with Forl (FB), indicating that the plant’s innate defense mechanisms were induced even without the presence of the pathogen. Therefore, such effects imply indirect interactions possibly via the induction of systemic acquired resistance (SAR) [44,45], given that SAR is mediated by pathways that are dependent on, but not only to, SA, JA, and ethylene [46,47]. Overall, the upregulation of genes involved in plant defense and plant growth may indicate defense priming that could explain the significant improvement in plant performance and Forl suppression observed in the presence of biosolid.

Elicitors of biosolid-mediated plant defenses include chemical compounds that are beneficial to the plant along with biosolid-induced microorganisms with potentially direct antagonistic effects towards Forl [4,35,36]. The potential induction of systemic acquired resistance in plants by compost mixtures, used as soil conditioners in plant growth substrates, has been demonstrated in several studies [48,49]. Therefore, it was hypothesized that these indirect plant defense mechanisms could also be induced by other biodegradable and non-composted materials, such as the anaerobically digested biosolid used in the present study.

Enhanced tomato performance against Forl could also be attributed to different types of interactions between the phytopathogenic fungus and the beneficial to the plant soil microflora, which is induced by biosolid application. Such interactions include competition for nutrients, production of antifungal metabolites, parasitism, and enzymatic hydrolysis of fungal cell walls [50,51,52]. Although the interaction mechanisms between microbial communities and plants are very complex, intense microbial diversity usually has a beneficial effect on both plant diversity and their growth and productivity [48,53,54]. Most of the microorganisms in the soil and the rhizosphere develop beneficial and mutual symbiotic relationships with the plant and among other microorganisms [55].

The bacterial biodiversity observed in the biosolid-enriched treatments, seems to have had a beneficial effect on both growth of the tomato plants and protection against Forl. The relation of the relative abundance of bacterial groups and pathogen inhibition along with development of disease suppression in soils has been recently demonstrated [16,17]. Herein, four phyla of bacteria were identified in greater abundance in biosolid-enriched soils, namely *Chloroflexi* (class *Anaerolineae*), *Bacteroidetes* (class *Bacteroidia*), *Patescibacteria* (class *Saccharimonadia*), and *Synergistetes* (class *Synergistia*). Interestingly, *Clostridia* (phylum *Firmicutes*), were detected solely in biosolid-enriched treatments and increased with time. Several genera within the phylum *Firmicutes*, have been shown to positively affect disease suppressiveness of soil amendments, such as compost [56]. *Clostridia* occur mainly in the rhizosphere and perform beneficial functions for the plants, such as atmospheric nitrogen fixation, phosphate solubilization and the reduction of Fe^3+^ to the more readily available iron form Fe^2+^ [57]. The higher abundance of *Bacilli* class was increased at 72 h under control conditions, yet it was reduced in B, FB, and F treatments. Bacillus species have exhibited ability for plant growth promotion [58] and have also demonstrated the ability to excrete exopolysaccharides, biosurfactants, and chelating agents, which are important for the remediation of heavy metals from soils [59]. *Bacillus* species have also demonstrated broad functions, especially in various enzymatic activities [60], indicating a possible role in pathogen control [61,62]. Nevertheless, the reduced abundance over time in the biosolid-enriched treatments indicate that possibly the presence or absence of mineral fertilization affects the structure of the bacterial community in the soil [63]. A possible explanation could be the acidification of soil by higher NPK content [64] present in the sewage sludge [65] and therefore the biosolid applied herein [12].

Other phyla of anaerobic bacteria, such as *Patescibacteria* and *Synergistetes*, that were observed mainly in the biosolid enriched treatment (B) have been shown to contribute to the degradation of organic matter [66,67]. Bacterial phyla such as *Chloroflexi* (class *Anaerolineae*) thrive under anaerobic conditions and are considered to play an important role in the vital process of photosynthesis [68]. In addition, these bacteria can degrade a large number of organic compounds and producing acetic acid [69,70], which has been shown to stimulate plant growth and inhibit the growth of pathogenic fungi [71,72].

The phylum *Proteobacteria* has a key role in anaerobic digestion by metabolizing volatile fatty acids [73]. In addition, bacteria in this phylum are known to remove a broad range of synthetic, as well as natural organic pollutants [74,75]. Within this phylum, the class *Alphaproteobacteria* were in higher abundance in C and F treatments compared to the biosolid-enriched ones, whereas *Gammaproteobacteria* were in higher abundance in B (increased over time) and FB (decreased over time) treatments. This indicates that different classes of the same phylum are affected differently by the addition of biosolid in the soils and in response to Forl, and further supports the evidence that consortia of beneficial microorganisms, rather than specific taxa, may drive disease suppression and lead to plant protection [76].

Similarly, we observed changes in abundance of *Actinobacteria*, *Firmicutes*, and *Acidobacteria* with time among the different treatments (Table S1). The higher abundance of the classes *Actinobacteria* and *Acidobacteria* have been associated with antagonistic activity toward several phytopathogens, such as *Fusarium* [77], and with suppressiveness in compost [78]. Additionally, high abundance in members of bacterial phyla, such as *Actinobacteria*, *Firmicutes*, and *Acidobacteria*, have been shown to directly antagonize pathogens through various mechanisms [17]. The example of *Firmicutes* abundance being increased over time under control (C) and Forl inoculation with biosolid-enriched soil (FB) treatment, remaining unchanged in the B treatment, and being reduced in the Forl inoculation (F) treatment indicates not only the presence, but also the change of abundance over time is significant. Additionally, *Acidobacteria* decreased in the B, F, and FB treatments and *Actinobacteria* increased in F, decreased in B, and remained unchanged in FB treatments. On the other hand, the abundance of *Actinobacteria*, *Firmicutes*, and *Acidobacteria* in the control treatment, where plants and microorganisms in the rhizosphere were undisturbed, was increased. These observations suggest that: (i) possibly different consortia of beneficial microorganisms, rather than specific species, may provide plant protection against Forl by suppressing the pathogen, which is in accordance with other studies [21,79], and (ii) the relative change (in the concept of increase or decrease) in abundance of these consortia over time may also play a regulatory role in the biocontrol of Forl.

Functional analysis was further performed to acquire information about the potential community functions. Different pathways associated with various aspects of metabolism were activated in biosolid-enriched (B and FB) compared to the non-enriched (C and F) treatments, indicating the impact of biosolid application on various microbial functions. For instance, in the secondary metabolism, vitamin biosynthesis pathway and especially folate biosynthesis pathway were activated in biosolid enriched soils. Potentially, the abundance of nutrients in the biosolid-enriched soil may have enhanced the biosynthesis of plant secondary metabolites, such as folates (reviewed by Kołton et al. [80]). Interestingly, studies have highlighted the importance of folates in inducing plant tolerance to several biotic and abiotic stresses [81,82]. Folates in plants are involved in redox homeostasis, physiological processes, epigenetic regulation, cell proliferation, and mitochondrial respiration, as well as photosynthesis [80,83,84], and have been shown to have antifungal functions [85].

The menaquinone (Vitamin K2) biosynthesis pathway was also activated in B and BF treatments. Menaquinones are involved in bacterial electron transport and in sensing environmental changes such as alterations in redox state; they have also been implicated in sporulation and proper regulation of cytochrome formation in all Gram-positive bacteria and anaerobically respiring Gram-negative bacteria [86,87,88]. In plants, vitamin K functions as a priming agent against biotic and abiotic stresses given its redox properties. Menadione (pro-vitamin K) was found to induce resistance by priming in Arabidopsis against the virulent strain *Pseudomonas syringae* pv. tomato DC3000, with more than two-fold PR1 expression in MSB-pretreated plants as compared to non-treated plants [89]. Similarly, in our study, an increase in PR1-a expression by 3.76-fold was observed 72 h in the B treatment, but not in the BF treatment, indicating the potential priming effect of biosolid in tomato plants.

The autotrophic CO_2_ fixation pathway found in the biosolid-enriched treatments indicated the presence of autotrophic microorganisms, which contribute significantly to CO_2_ fixation in the soil carbon sink of agricultural soils [90]. Other pathways activated in the biosolid-enriched treatments include the carbohydrate biosynthesis (gluconeogenesis) indicative of sugar synthesis [91], which is the primary source of energy for all eukaryotic organisms [92]. Specifically, in plants, they are involved in most metabolic and signaling pathways controlling growth, development, and fitness [92]. Additionally, in the biosolid-enriched treatments, sequences were attributed to the nitrogen metabolism pathway which, according to Jacoby et al. [93], when active in the rhizosphere, is an indicator that the microbiome plays an important role in mediating plant nutrition. Recent studies have shown that amino acids play a key role in plant root growth and microbial colonization, symbiotic interactions, and pathogenesis in the rhizosphere [94]. Amino acids are considered a key intermediary in the soil nitrogen cycle, and function as carbon and nitrogen sources for both microorganisms and plants, in synthesis and regulation of auxin activity and biofilm formation and disassembly [95]. Therefore, the nucleic acid processing for protein synthesis pathway, which was found active in biosolid-enriched soils, could be an indicator of intrinsic amino acid biosynthesis.

Control and Forl treatments shared common pathways that were active under such conditions including: aerobic respiration and the related TCA cycle pathways, important processes in the global carbon cycle and of crucial importance in the partitioning of energy in soil [96]; the tetrapyrrole biosynthesis (TBS) and heme b biosynthesis pathways, important for oxidative and energy metabolism in a variety of biological functions, such as gas transport, respiration, and nitrite and sulphite reduction [97]; and also in chlorophyll synthesis in plants and algae [98]. In S-adenosyl-L-methionine (SAM) biosynthesis, SAM functions as a methyl donor and plays a key role in antibiotic production [99], and inhibits sporulation and cellular differentiation in *Streptomyces* spp., *Bacillus subtilis*, and *Saccharomyces cerevisiae* [100]. Other functions detected include the sugar nucleotide biosynthesis and sugar degradation (galactose). It has been previously shown that galactose metabolism plays a central role in biofilm formation by *B. subtilis* and other bacteria [101]. Nevertheless, Forl inoculation treatment also showed higher abundance of sequences attributed to Carboxylate (sugar acid) and secondary metabolism (sugar derivative; sulfoquinovose) degradation. Sulfoquinovose biosynthesis is largely conserved within plants, algae, and photosynthetic bacteria and plays a major role in the global biogeochemical sulfur cycle by serving as a sulfur reservoir that can be mobilized in the early stages of sulfur starvation [102]. Nevertheless, a sulfoglycolytic pathway is being employed by a diverse collection of bacterial species, such as γ-*Proteobacteria*, as well as α- and β-*Proteobacteria* [103].

Overall, based on the results of this research, the biosolid application seems to result in a successful priming of tomato plants inducing resistance mechanisms against Forl. This effect was also associated with the microbiome diversity in the biosolid-enriched treatments and the changes in abundance with time in response to Forl. Organic amendments, such as green manures, stable manures, and composts, have long been recognized to facilitate biological control within the context of bacterial communities [104]. Microbe-microbe associations and microbe–plant interactions are important in the context of pathogen inhibition via direct antagonism and mediating processes involved in nutrient dynamics [105,106]. Therefore, the use of biosolid as a soil amendment had a positive effect not only on plant health, but also on the bacterial diversity, relative abundance and predicted soil functioning, toward enhancing tomato resistance against Forl.

## 4. Materials and Methods

### 4.1. Culture of Fusarium oxysporum f. sp. radicis-lycopersici and Inoculum Preparation

A virulent strain of Forl provided by the Institute Biology, Leiden University, Leiden, the Netherlands and deposited in the Centraalbureau voor Schimmelcultures, the Netherlands (CBS 101587), was used for artificial inoculations of tomato plants. The fungus was routinely kept on potato dextrose agar (PDA; Lab M, Lancashire, UK), at 4 °C and was often inoculated on a surface-sterilized tomato fruit and re-isolated on PDA to maintain its virulence. For inoculum preparation, Forl was grown in Czapek Dox Broth (Ducheta Biochemie, Haarlem, The Netherlands) as described in Kamou et al. [3]. Conidia were separated from mycelium, washed with sterile distilled water, and adjusted to 10^6^ spores mL^−1^, as previously described by Giannakis et al. [12].

### 4.2. In Vitro Growth and Sporulation of Fusarium oxysporum f. sp. radicis-lycopersici in the Presence of Biosolid Leachates

Anaerobically digested and dehydrated sludge [12] was subjected to the one-step static leaching method ΕΝ 12457-2 as described in Giannakis et al. [107] and was sterilized by filtration through 0.2 μm pore antimicrobial filters (Whatman Puradisk^TM^, Buckinghamshire, UK) before incorporation into PDA at a temperature of 40 °C, just before pouring plates. Growth substrates of biosolid leachates and PDA mixtures were prepared at concentrations of 0% (control), 2%, 5%, and 10% *v*/*v* in Petri dishes. Forl discs, 4 mm in diameter, taken from the periphery of a fresh colony on PDA, were used to inoculate the different mixtures of PDA-biosolid leachates and cultures were incubated for 6 days. After incubation, fungal growth was expressed as colony diameter in centimeters, as described by Bardas et al. [108]. More specifically, two vertical lines were drawn at the bottom of each plate with an intersection point at the center of the inoculation point. The diameter of the colony was measured on each line and an average value of 10 measurements derived from 5 replications (Petri dishes) was calculated.

Sporulation of the fungus in the PDA-biosolid leachates media of different concentrations was assessed as follows: 10 pieces of 0.5 cm^2^, from the periphery of each colony, were added to 20 mL sterile distilled water in screw-capped sterile tubes. Conidia were detached from mycelium by vortexing each tube for 1 min and were harvested by filtering through sterile Miracloth (Calbiochem, San Diego, CA, USA) followed by a 10^3^-fold dilution with sterile distilled water under sterile conditions. Then, 100 μL of the diluted mixtures were placed on PDA plates and incubated for 2 days at 20–25 °C. The number of single spore colonies obtained was counted and the initial number of conidia per cm^2^ of colony was calculated for the different media. The fungal growth and sporulation assays were repeated 5 times.

### 4.3. In Planta Experiment and Relative Gene Expression Analysis in Tomato Leaves

#### 4.3.1. Substrate Preparation, Tomato Plant Growth, and Inoculation Procedure

For the *in planta* experiment, four different substrates were prepared from peat and clay soil with two levels of biosolid (absence or presence), to which two levels of inoculum were applied; without (−) or with (+) Forl, as described by Giannakis et al. [12]. Specifically, growth substrates were: (i) peat and clay soil 1:1 (*w*/*w*) (treatment C), (ii) peat and clay soil 1:1 (*w*/*w*), supplemented with biosolid 2% (*w*/*w*) (treatment B), (iii) peat and clay soil 1:1 (*w*/*w*) inoculated with Forl (treatment F), and (iv) peat and clay soil 1:1 (*w*/*w*) supplemented with biosolid 2% (*w*/*w*) and inoculated with Forl (treatment FB). Clay soil was characterized as 40% silt, 30% clay, 30% sand, 2.5% organic matter, and pH 7.9. Inoculations and plant growth conditions were as described by Giannakis et al. [12]. Specifically, Forl inoculum, prepared as described above, was mixed thoroughly into the substrates at a 1/10 volume ratio, to a final concentration of 10^5^ spores g^−1^ substrate. Non-inoculated substrates were mixed with water. Tomato seedlings of the cv. ACE 55 at the two-leaf stage were transplanted in the different substrates in 0.1 L pots. All plants were placed in the same growth chamber and were grown under controlled conditions at 20–25 °C with 14/10 h photoperiod light/dark, respectively, and 60% RH.

#### 4.3.2. Gene Expression Analysis

Gene expression analysis was monitored at 12, 24, 48, and 72 h in leaves of tomato after transplantation and exposure to biosolids and Forl. The relative gene expression analysis of *GLUA*, *CHI3*, *PR1-a*, *LOX*, and *AOC* genes (Table 5), associated with enhanced resistance against pathogen infection in tomato plants, was performed using the reverse-transcription quantitative polymerase chain reaction (RT-qPCR). Total RNA from tomato leaves was extracted using the Monarch Total RNA miniprep kit (New England Biolabs Inc., Ipswich, MA, USA) in three biological replications at each time point and treatment. RNA quality and quantity were assessed using the UV-Vis Spectrophotometer Q5000 (Quawell Technology Inc., San Jose, CA, USA) and optically with gel electrophoresis in 1.5% agarose gel. Pooling of biological replicates was performed equimolarly with 1 μg per replicate at a working concentration of 100 ng μL^−1^.

The cDNA was prepared with 1 μg pooled RNA using the PrimeScript™ Reverse Transcriptase kit (TAKARA BIO Inc., Kusatsu, Shiga, Japan) and random hexamers according to the manufacturers’ instructions. Relative gene expression was assessed by real-time quantitative reverse transcriptase PCR (RT-qPCR) performed on a Rotor-Gene 6000 real-time 5-Plex HRM PCR Thermocycler (Corbett Research, Sydney, Australia) using the Rotor-Gene Q software version 2.0.2 (Corbett Life Science, Cambridge, UK) and melt curve analysis. The reaction mixtures were prepared in a total volume of 20 μL per reaction consisting of 50 ng cDNA, 1× PCR buffer, 0.5 μM forward and reverse primers, 0.2 mM dNTPs, 1.5 mM SYTO™ 9 Green Fluorescent Nucleic Acid Stain (Invitrogen, Eugene, OR, USA), and 1 U Kapa Taq DNA polymerase (Kapa Biosystems, Wilmington, MA, USA). The amplification was performed according to the following thermal cycling conditions: initial denaturation at 95 °C for 2 min, followed by 35 cycles of 95 °C for 10 s, 54 °C for 25 s, and 72 °C for 30 s. Fluorescence was acquired at the end of each PCR cycle. Melting curve analysis was performed at temperature range between 65–95 °C and in increments of 0.3 °C every 2 s; fluorescence was measured at the end of each increment step.

The tomato gene-specific primers (Table 5) *GLUA* [35], *CHI3* [35,37], *PR1*-*a*, *LOX* [35], and *AOC* [7,36,109] were used for the relative gene expression analysis. The housekeeping genes encoding for actin (*β-actin*) [35], mitochondrion cytochrome oxidase subunit I (*CyOXID*) [110], and Glyceraldehyde-3-phosphate-dehydrogenase (*Gapdh*) [51] were used as reference genes for normalization in tomato. Data analysis was carried out with relative quantification in three technical replicates for each pool, using the 2^−ΔΔCT^ method [111], and data normalization was achieved using the expression levels of the reference genes.

### 4.4. Characterization of the Soil Substrate Microbiome Using 16S Sequencing

Soil substrate samples near the roots were collected from the same pots that the leaf tissue was collected, at 12 and 72 h after treatment application, in 3 biological replications. The total microbial genomic DNA was extracted with the NucleoSpin Soil, Mini kit (Macherey-Nagel GmbH & Co.KG., Düren, Germany). The DNA quantity and quality were assessed using the UV-Vis Spectrophotometer Q5000 (Quawell Technology Inc., San Jose, CA, USA). The 16S rRNA libraries were constructed after amplification of the 16S subunit of the prokaryotic ribosomal 16S RNA gene (16S rRNA), using primers that amplify between the V3 and V4 regions of the gene for each sample. The 2 × 300 bp paired-end reads were generated using the Illumina MiSeq platform (Illumina Inc., San Diego, CA, USA).

For the identification and quantification of the detected microorganisms, as well as the comparison between the samples, bioinformatics analysis was performed using Mothur programs (1.44.1) and R packages (4.0.3) [112], Phyloseq (1.34.0), ggplot2 (3.3.2), DESeq2 (1.28.1), and vegan (2.5.6). The raw sequencing data were processed to remove low-quality sequences as well as DNA adapters. Pure sequences were grouped into Operational Taxonomic Unit (OTUs) with a sequence similarity rate of 97%. The classification of OTUs was characterized by SILVA multiple sequencing (SILVA alignment Release 132, 8517 bacteria, 147 archaea, and 2516 eukarya sequences). The Mothur analysis was performed following the Standard Operating Procedure (SOP) for the Mothur Metagenomics analysis as described in Schloss et al. [113].

Variation of microbial communities within a single soil substrate or between the soil substrates was found using alpha and beta diversity, respectively. More specifically, the α-diversity was evaluated by rarefaction curves measuring the Shannon, Chao1, Abundance-based Coverage Estimator (ACE), and Simpson diversity indices. The β-diversity was assessed by non-metric multidimensional scaling (NMDS) to define the structure of the microbiome. Canonical correspondence analysis (CCA) was used to relate species abundance to the treatment and time variables. A graphic interpretation of the main principal axes by tri-plot on the two dimensions was obtained with the Phyloseq software. The functional potential of the detected microbial communities was predicted based on the 16s rRNA marker using the PICRUSt software (2.3.0_b) [114]. The functional prediction was based on the unique OTU sequences and the biom file produced by Mothur (1.44.11). The analysis returned the relative abundance of the predicted EC codes and their related pathways description. The statistical analysis and final visualization of the results were performed using the Statistical Analysis of Metagenomic Profiles (STAMP) software (2.1.3) [115].

### 4.5. Statistical Analysis

Data from fungal growth analysis and sporulation assay (n = 4) and relative gene expression analysis (n = 3) were subjected to analysis of variance (ANOVA) based on the completely randomized design (CRD). Differences among the treatments were assessed using Tukey’s post hoc test at a significance level predetermined at *p* ≤ 0.05. All statistical analyses were performed using SPSS Statistics for Windows, v.25 (IBM Corp., Armonk, NY, USA). For the functional analysis, the Statistical Analysis of Metagenomic Profiles (STAMP) [115] software was used to provide a statistical view of differences in abundant features. The data were subjected to analysis of variance (ANOVA) and differences among the treatments were assessed using Tukey–Kramer post hoc test at a significance level at *p* ≤ 0.05.

## 5. Conclusions

This is the first attempt to better understand the plant–microbiome interactions in the presence of biosolid application and the biocontrol mechanism against Forl in tomato plants. More specifically, the effect of biosolid application on the biocontrol of Forl was investigated based on the enhanced plant resistance measured as expression of pathogen-response genes and the pathogen suppression in the context of soil microbiome diversity, abundance, and predicted functions. Plants and rhizosphere microbiome share complex interactions required for optimal root and soil functioning. When this balance is disturbed, changes occur in microbial communities, soil functioning, and soil abiotic properties interactively [106], shaping the resistance potential of plants and the biocontrol of the pathogen. Our results suggest that biosolid application alters microbial diversity and the predicted soil functioning, along with the relative abundance of specific phyla and classes, as a proxy for disease suppression. Further research is required to identify the biochemical and molecular mechanisms of the priming effect induced by the biosolid and specific functional genes associated with bacterial consortia as biological indicators for the identification of the biocontrol potential of biosolid application.

## Figures and Tables

**Table 1:** Mean number of *Fusarium oxysporum* f. sp. *radicis-lycopersici* conidia produced per cm^2^ of colony at different biosolid leachate–PDA concentrations in PDA. Different letters indicate statistically significant differences between the treatments according to Tukey’s post hoc test (*p* < 0.05).

Leachate Concentration (%)	Mean Initial Number of Conidia Per cm^2^	Tukey’s Post Hoc Test
0	(176 ± 119) × 10^3^	b
2	(184 ± 166) × 10^3^	b
5	(404 ± 119) × 10^3^	b
10	(808 ± 314) × 10^3^	a

**Table 2:** Proportion of inertia explained by constrained and unconstrained ordination.

	Inertia	Proportion
Total	3.9869	1
Constrained	0.9103	0.2283
Unconstrained	3.0766	0.7717

**Table 3:** Accumulated constrained eigenvalues.

Importance of Components:	CCA1	CCA2	CCA3	CCA4
Eigenvalue	0.4155	0.1738	0.1681	0.1529
Proportion Explained	0.4565	0.1909	0.1847	0.1679
Cumulative Proportion	0.4565	0.6474	0.8321	1

**Table 4:** Biplot scores for constraining variables.

Factors	CCA1	CCA2
Time 72 h	−0.1404	−0.7527
Treatment B	−0.5594	−0.2868
Treatment F	0.5453	0.3655
Treatment FB	−0.5831	0.3844

**Table 5:** Primers used in RT-qPCR of the 5 genes (*GLUA, CHI3, PR1-a, LOX,* and *AOC*) associated with response mechanisms to pathogens and the 3 housekeeping genes (*β-actin, CyOXID,* and *Gapdh*).

Gene		Gene Sequence	Encoding Protein	Defense Pathway
*GLUA*	F	GTCTCAACCGCGACATATT	PR-2 (β-1,3 glucanase, basic type)	SA signaling pathway
	R	CACAAGGGCATCGAAAAGAT		
*CHI3*	F	TGCAGGAACATTCACTGGAG	PR-3 (Chitinase)	JA/ETH signaling pathway
	R	TAACGTTGTGGCATGATGGT		
*PR1-a*	F	TCTTGTGAGGCCCAAAATTC	PR-1 (acidic type)	SA signaling pathway
	R	TAGTCTGGCCTCTCGGACA		
*LOX*	F	CCTGAAATCTATGGCCCTCA	Lipoxygenase	ETH signaling pathway
	R	ATGGGCTTAAGTGTGCCAAC		
*AOC*	F	CTCGGAGATCTTGTCCCCTTT	Allene oxide cyclase	JA/ETH signaling pathway
	R	CTCCTTTCTTCTCTTCTTCGTGCT		
*β-actin*	F	GAAATAGCATAAGATGGAGACG	Actin	Reference gene
	R	ATACCCACCATCACACCAGTAT		
*CyOXID*	F	TGGTAATTGGTCTGTTCCGATT	Cytochrome oxidase subunit I	Reference gene
	R	TGGAGGCAACAACCAGAATG		
*Gapdh*	F	GAAATGCATCTTGCACTACCAACTGTCTTGC	Glyceraldehyde-3-phosphate-dehydrogenase	Reference gene
	R	CTGTGAGTAACCCCATTCATTATCATACCAAGC		

## Data Availability

Data is contained within the article or Supplementary Materials.

## Supplementary Materials

The following are available online at https://www.mdpi.com/article/10.3390/plants10122789/s1, Figure S1: Rank abundance curves. Abundances of the top 100 OTUs for bacterial communities in: A. Between two time points at 12 and 72 h after inoculation with *Fusarium oxysporum* f. sp. *radicis-lycopersici* and biosolid application. B. amongst the four different treatments control (C), biosolid (B), Forl (F), and Forl and biosolid (FB), Figure S2: Principal Component Analysis (PCA) of the functional diversity for the four different treatments control (C), biosolid application (B), Forl inoculation (F), and Forl inoculation + biosolid application (FB) at 12 and 72 h post inoculation and biosolid application. The different treatments are depicted with different colors and shapes, Figure S3: Post hoc plots for the predicted pathways demonstrating greater abundance of sequences (%) in biosolid-enriched treatments compared to the Control (C) and Forl inoculation + biosolid application (FB) treatments, indicating: (i) the mean proportion of sequences within each treatment, (ii) the difference in mean proportions for each pair of treatments, and (iii) a *p*-value indicating whether the mean proportion is equal for a given pair of treatments. The analysis was performed in the STAMP software using ANOVA with *p*-value ≤ 0.05 and effect size >0.9, Table S1: Relative abundance (%) of bacterial phyla at rate greater than 1%, for the treatments C, B, F, and FB at 12 and 72 h after inoculation with *Fusarium oxysporum* f. sp. *radicis-lycopersici*. Value is the pooled mean of three replicates, Table S2: Relative abundance (%) of bacterial classes at rate greater than 1%, for the treatments C, B, F, and FB at 12 and 72 h after inoculation with *Fusarium oxysporum* f. sp. *radicis-lycopersici*. Value is the pooled mean of three replicates, Table S3: Multiple group statistics table for the predicted functional diversity in the different treatments: Control (C), Biosolid application (B), Forl inoculation (F), and Forl inoculation + biosolid application (FB) based on the abundances of sequences associated with specific metabolic pathways. The analysis was performed in the STAMP software using ANOVA with *p*-value ≤ 0.05 and effect size >0.9. The order of pathways is according to the abundance (%) as indicated in Figure 7.
