# Supplementary material for: Biosolid-Amended Soil Enhances Defense Responses in Tomato Based on Metagenomic Profile and Expression of Pathogenesis-Related Genes
Source: Plants (Basel). 2021 Dec 16;10(12):2789. doi: 10.3390/plants10122789 (PMC8709368; doi:10.3390/plants10122789)
Supplement: Supplementary file 1 [file plants-10-02789-s001.zip › Supplementary material.pdf]

Biosolid-amended soil enhances defense responses in tomato based on metagenomic profile and expression of pathogenesis-related genes

Evangelia Stavridou[1,2], Ioannis Giannakis[3], Ioanna Karamichali[2], Nathalie N. Kamou[4], George Lagiotis[2], Panagiotis Madesis[2,5], Christina Emmanouil[6], Athanasios Kungolos[3], Irini Nianiou-Obeidat[2*] and Anastasia Lagopodi[4*]

1 Institute of Applied Biosciences, Centre for Research and Technology Hellas, Thermi, Thessaloniki, Greece;
2 Laboratory of Genetics and Plant Breeding, School of Agriculture, Forestry and Natural Environment, Aristotle University of Thessaloniki, Thessaloniki, Greece; nianiou@agro.auth.gr
3 School of Civil Engineering, Aristotle University of Thessaloniki, Thessaloniki, Greece;
4 Laboratory of Plant Pathology, School of Agriculture, Forestry and Natural Environment, Aristotle University of Thessaloniki, Thessaloniki, Greece; lagopodi@agro.auth.gr
5 Laboratory of Molecular Biology of Plants, School of Agricultural Sciences, University of Thessaly, Volos, Greece;
6 School of Spatial Planning and Development, Aristotle University of Thessaloniki, 54124 Thessaloniki, Greece;

* Correspondence: A.L.: lagopodi@agro.auth.gr; I.N.O.: nianiou@agro.auth.gr

Table S1. Relative abundance (%) of bacterial phyla at rate greater than 1%, for the treatments C, B, F and FB at 12 and 72 h after inoculation with *Fusarium oxysporum* f. sp. *radicis-lycopersici*. Value is the pooled mean of three replicates.

**Treatments**	**B**		**C**		**F**		**FB**	
**Phylum (%)**	**12h**	**72h**	**12h**	**72h**	**12h**	**72h**	**12h**	**72h**
Acidobacteria	8.5	7.1	16.3	14.7	14.1	12.0	8.1	7.0
Actinobacteria	11.1	9.6	11.9	12.7	12.4	13.2	8.9	8.6
Bacteria_unclassified	6.8	7.3	7.3	8.2	7.0	7.9	6.4	6.6
Bacteroidetes	11.5	12.0	9.7	8.5	8.8	9.8	11.4	13.3
Chloroflexi	7.2	9.7	3.1	3.2	2.4	2.7	6.0	11.1
Cyanobacteria	-	-	-	-	1.1	-	-	-
Firmicutes	2.7	2.7	2.2	3.2	3.6	2.6	1.7	3.3
Gemmatimonadetes	1.8	1.5	3.6	2.9	2.7	2.8	1.9	1.3
Patescibacteria	2.1	1.9	-	-	-	-	1.3	2.5
Planctomycetes	7.2	6.4	6.4	7.7	7.6	6.8	7.1	5.3
Proteobacteria	34.6	35.2	33.9	33.6	35.8	35.9	41.1	34.4
Synergistetes	1.5	1.4	-	-	-	-	-	1.1
Verrucomicrobia	3.8	2.9	4.2	3.8	4.0	5.0	3.7	3.2
Total %	98.7	97.7	98.6	98.6	99.5	98.7	96.2	97.9

Table S2. Relative abundance (%) of bacterial classes at rate greater than 1%, for the treatments C, B, F and FB at 12 and 72 h after inoculation with *Fusarium oxysporum* f. sp. *radicis-lycopersici*. Value is the pooled mean of three replicates.

**Treatments**		**B**		**C**		**F**		**FB**	
**Phylum**	**Class (%)**	**12h**	**72h**	**12h**	**72h**	**12h**	**72h**	**12h**	**72h**
Actinobacteria	Acidimicrobiia	2.6	2.5	1.7	1.5	1.5	1.5	1.8	2.6
	Actinobacteria	5.1	4.9	5.8	6.9	7.1	7.5	4.4	4.2
	Actinobacteria_unclassified	-	-	2	1.1	-	1.1	-	-
	Thermoleophilia	2.4	1.7	2.7	2.6	2.6	2.8	1.7	1.3
Acidobacteria	Acidobacteriia	2.6	2.2	5.3	4.7	4.8	4.5	2.4	1.9
	Blastocatellia_(Subgroup_4)	1.7	1.47	3.8	3.2	2.7	2.1	1.7	1.5
	Subgroup_6	3.1	2.9	6.2	5.9	5.9	4.8	3.3	2.7
Bacteria_unclassified	Bacteria_unclassified	6.8	7.3	7.3	8.2	7	7.9	6.4	6.6
Bacteroidetes	Bacteroidia	11.1	11.4	9.6	8.5	8.7	9.8	11.1	12.6
Chloroflexi	Anaerolineae	6.1	8.9	1.5	1.6	1.5	1.3	5	10.3
Cyanobacteria	Oxyphotobacteria	-	-	-	-	1.1	-	-	-
Firmicutes	Bacilli	1.4	-	1.8	2.45	2.9	2	1	1.5
	Clostridia	1.4	1.5	-	-	-	-	1.1	1.8
Gemmatimonadetes	Gemmatimonadetes	1.4	1.3	2.6	2.3	2.2	2.3	1.5	1.5
	S0134_terrestrial_group	-	-	1.1	-	-	-	-	-
Proteobacteria	Alphaproteobacteria	15.4	15.2	18.4	19	18.2	19.5	17.7	14.8
	Deltaproteobacteria	3.3	3.2	2.3	2.2	1.9	2.1	3.7	3.3
	Gammaproteobacteria	15.8	16.6	13.1	12.2	15.7	14.1	19.5	16.1
Patescibacteria	Saccharimonadia	1	1.1	-	-	-	-	-	1.3
Planctomycetes	Phycisphaerae	2.4	2.1	3.3	3	3.5	3	2.8	1.4
	Planctomycetacia	4.6	4.2	2.9	4.5	4	3.8	4.1	3.9
Synergistetes	Synergistia	2	1.4	-	-	-	-	-	1.1
Verrucomicrobia	Verrucomicrobiae	3.8	2.9	4.2	3.8	4	5	3.7	3.3
**Total%**		94	92.7	95.6	93.6	95.3	95.1	92.9	93.7

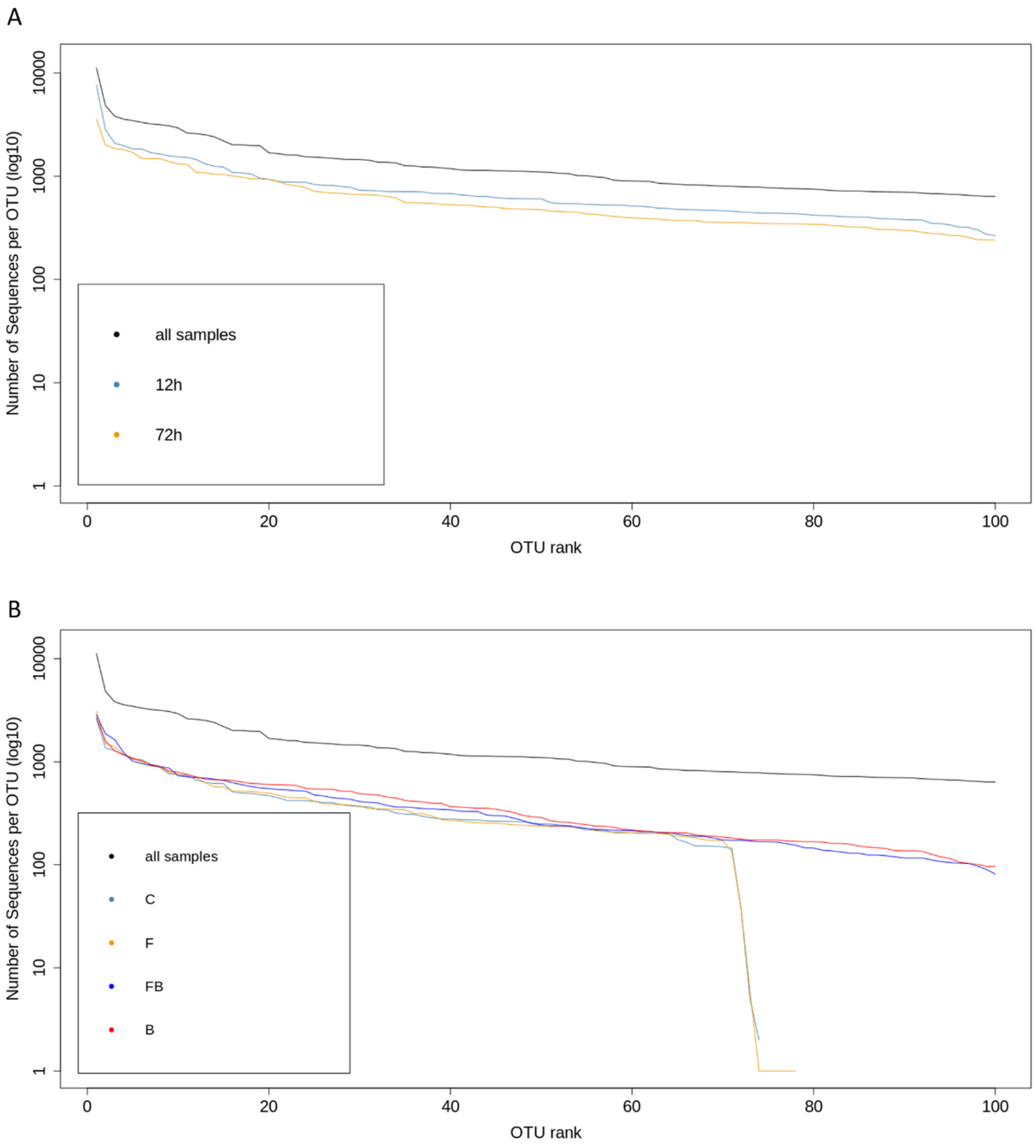

Figure S1. Rank abundance curves. Abundances of the top 100 OTUs for bacterial communities in: A. Between two time points at 12 and 72 h after inoculation with *Fusarium oxysporum* f. sp. *radicis-lycopersici* and biosolid application. B. amongst the four different treatments control (C), biosolid (B), (Forl) F and Forl and biosolid (FB).

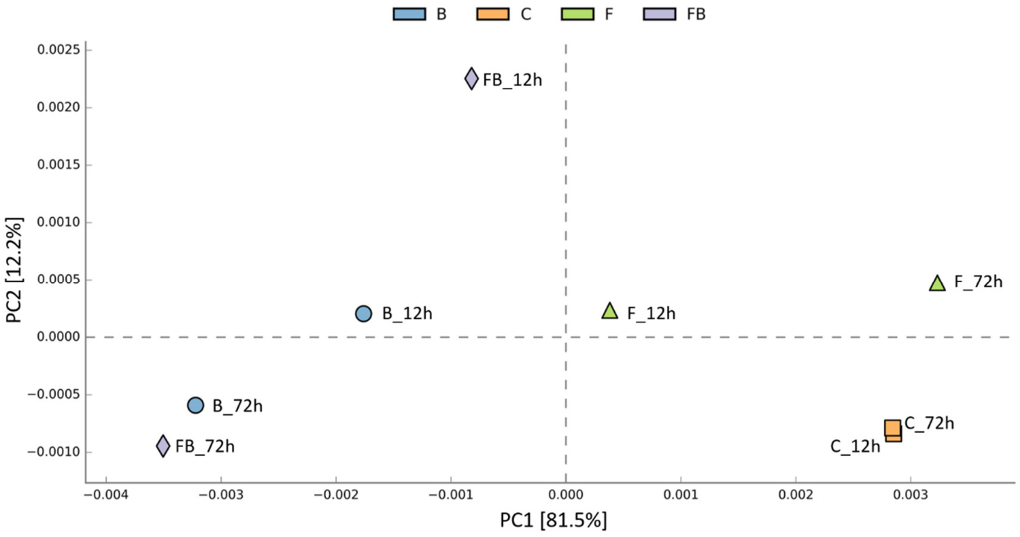

Figure S2. Principal Component Analysis (PCA) of the functional diversity for the four different treatments control (C), biosolid application (B), ForI inoculation (F), and ForI inoculation + biosolid application (FB) at 12 and 72 h post inoculation and biosolid application. The different treatments are depicted with different colours and shapes.

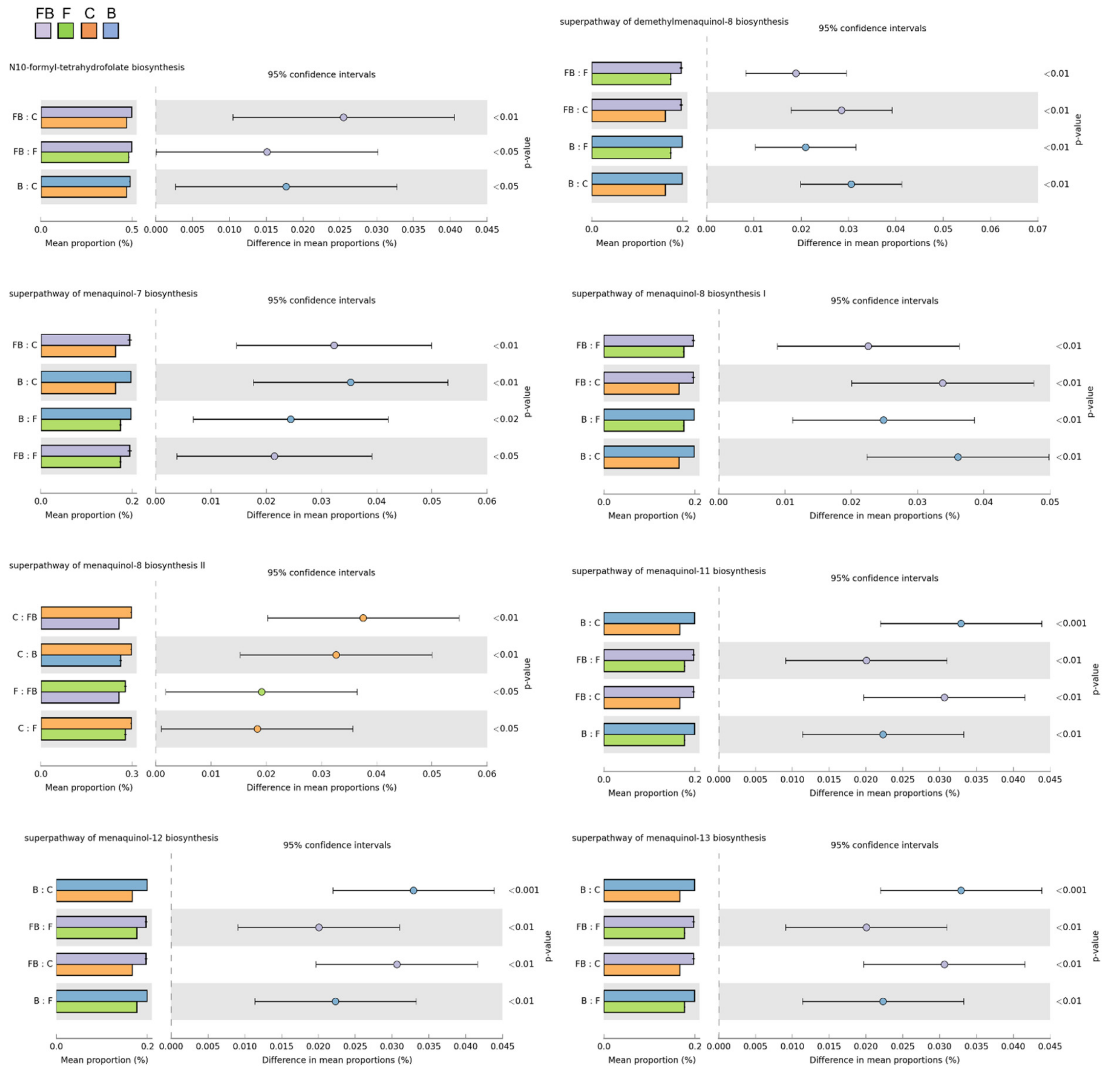

Figure S3. Post-hoc plots for the predicted pathways demonstrating greater abundance of sequences (%) in biosolid-enriched treatments compared to the Control (C) and ForI inoculation + biosolid application (FB) treatments, indicating: i) the mean proportion of sequences within each treatment, ii) the difference in mean proportions for each pair of treatments, and iii) a *p*-value indicating whether the mean proportion is equal for a given pair of treatments. The analysis was performed in the STAMP software using ANOVA with *p*-value ≤ .05 and effect size >0.9.
