# Supplementary figures and images for: Biosolid-Amended Soil Enhances Defense Responses in Tomato Based on Metagenomic Profile and Expression of Pathogenesis-Related Genes
Source: Plants (Basel). 2021 Dec 16;10(12):2789. doi: 10.3390/plants10122789 (PMC8709368; doi:10.3390/plants10122789)

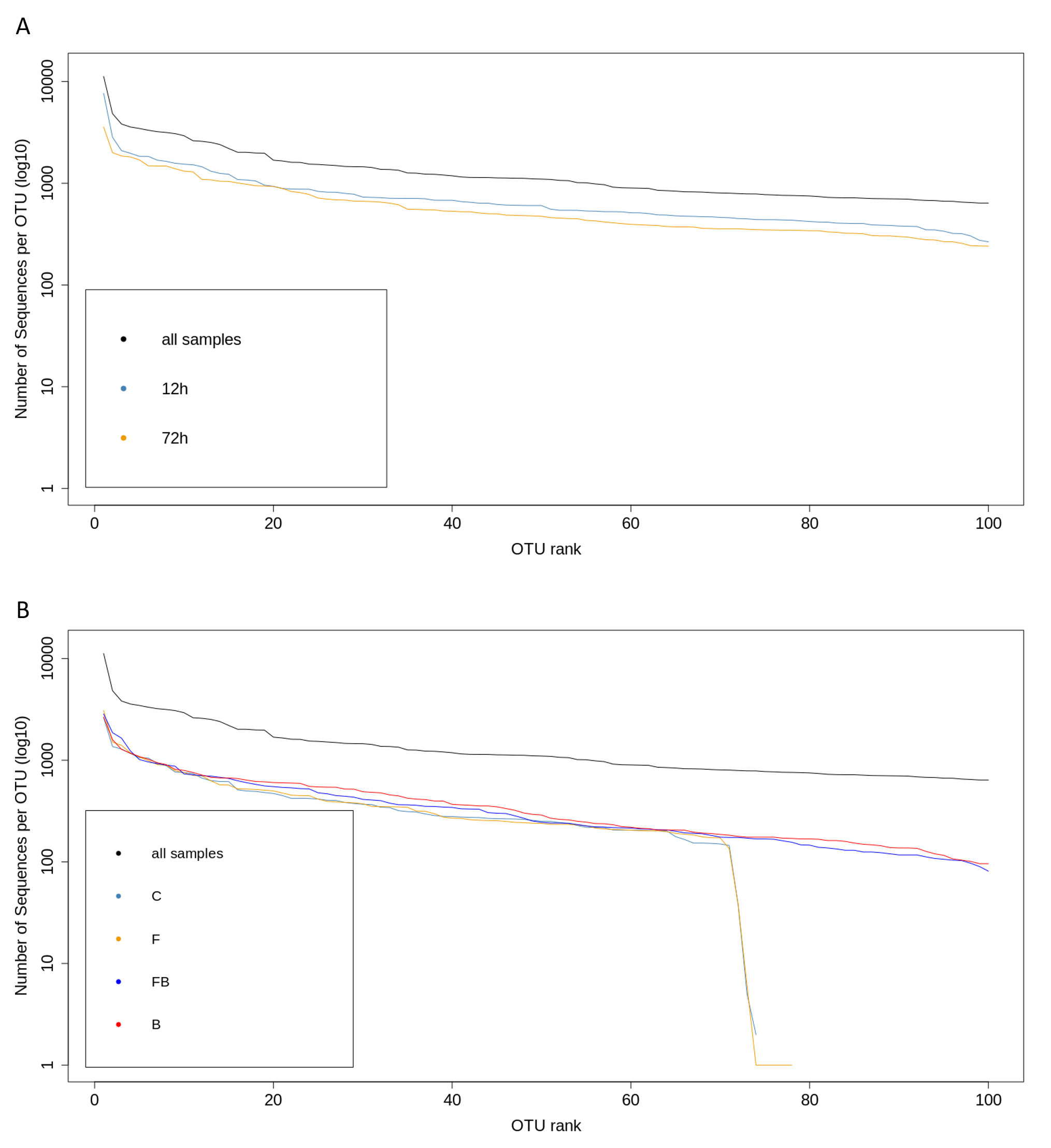

Supplement: Supplementary file 1 [file plants-10-02789-s001.zip › Figure S1.tif]

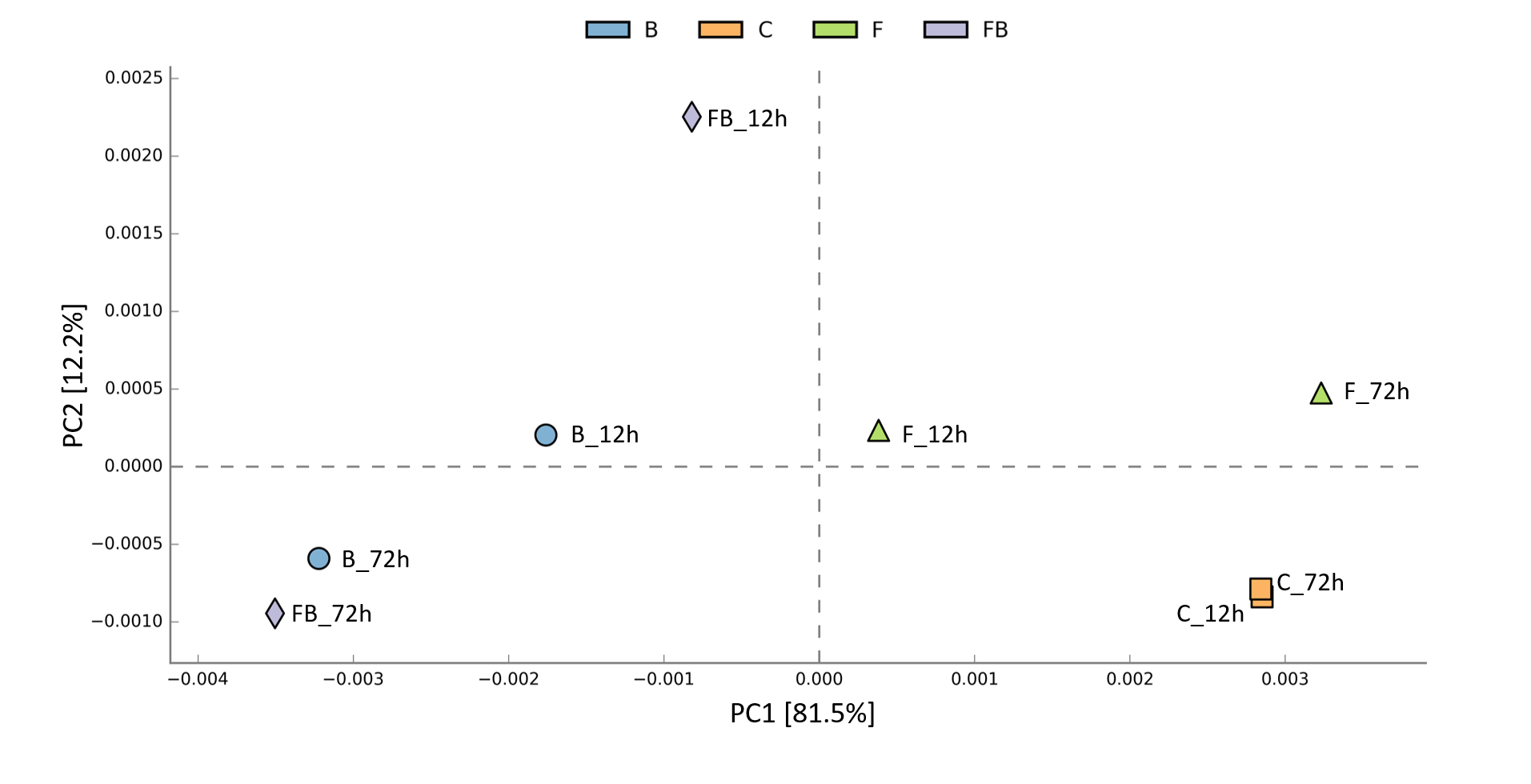

Supplement: Supplementary file 1 [file plants-10-02789-s001.zip › Figure S2.tif]

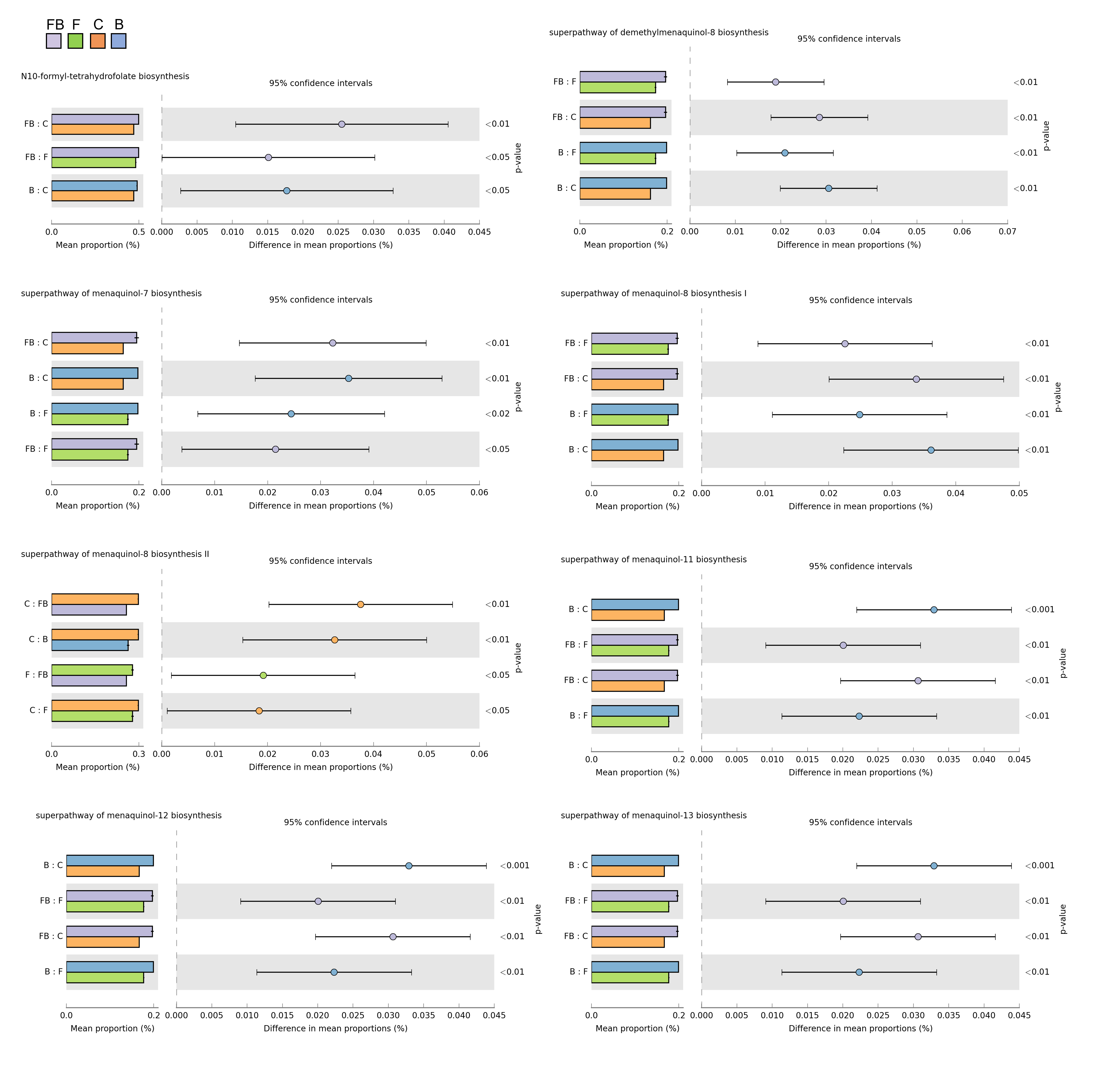

Supplement: Supplementary file 1 [file plants-10-02789-s001.zip › Figure S3.png]
